# Supplementary material for: The interactome of intact mitochondria by cross-linking mass spectrometry provides evidence for coexisting respiratory supercomplexes
Source: Mol Cell Proteomics. 2017 Dec 8;17(2):216–32. doi: 10.1074/mcp.RA117.000470 (PMC5795388; doi:10.1074/mcp.RA117.000470)
Supplement: Supplemental Data [file supp_RA117.000470_133922_0_supp_23978_fzffwf.zip › spectra_annotation/mito_DR_spectra_annotation/460-1-6-1-5-1.pdf]

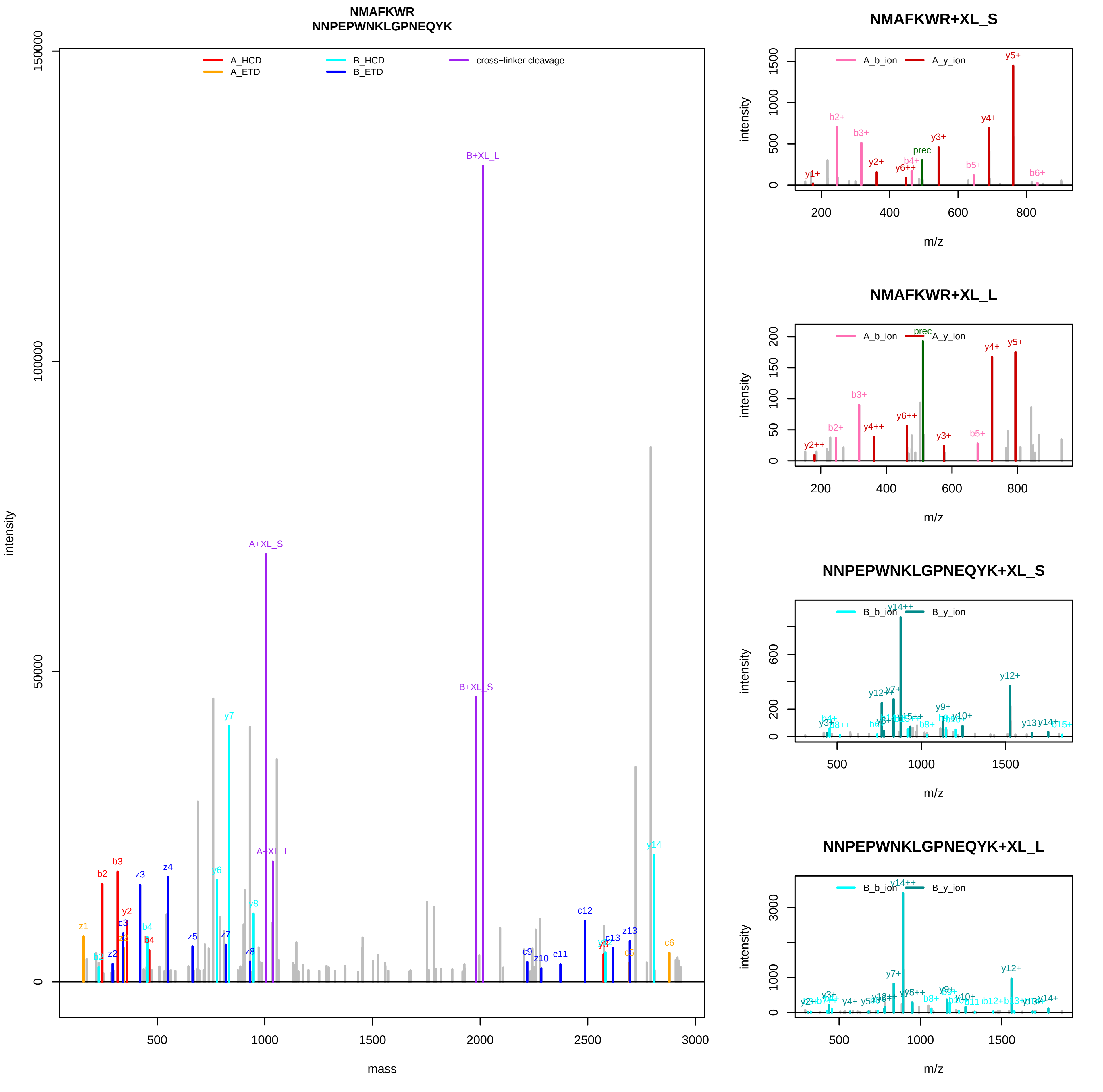

NMAFKWR
NNPEPWNKLGPNEQYK
A_HCD
A_ETD
B_HCD
B_ETD
cross−linker cleavage
intensity
mass
0
50000
100000
150000
500
1000
1500
2000
2500
3000
B+XL_L
A+XL_S
B+XL_S
A+XL_L
z1
b2
b3
y2
c3
z2
z3
b4
z4
z5
y6
y7
z7
z8
y8
c9
z10
c11
c12
y3
c13
z13
c5
c6
y14
NMAFKWR+XL_S
A_b_ion
A_y_ion
intensity
m/z
y1+
b2+
b3+
y2+
y6++
b4+
prec
y3+
b5+
y4+
y5+
b6+
NMAFKWR+XL_L
y2++
b2+
b3+
y4++
y6++
y3+
b5+
y4+
y5+
NNPEPWNKLGPNEQYK+XL_S
B_b_ion
B_y_ion
y14++
y12++
y7+
y9+
y10+
y12+
y13+
y14+
NNPEPWNKLGPNEQYK+XL_L
y14++
y7+
y9+
y10+
y12+
y14+
